# Screening for Chemical Contributions to Breast Cancer Risk: A Case Study for Chemical Safety Evaluation

**DOI:** 10.1289/ehp.1408337

**Published:** 2015-06-02

**Authors:** Megan R. Schwarzman, Janet M. Ackerman, Shanaz H. Dairkee, Suzanne E. Fenton, Dale Johnson, Kathleen M. Navarro, Gwendolyn Osborne, Ruthann A. Rudel, Gina M. Solomon, Lauren Zeise, Sarah Janssen

**Affiliations:** 1 Center for Occupational and Environmental Health, School of Public Health, University of California, Berkeley, Berkeley, California, USA; 2 Silent Spring Institute, Newton, Massachusetts, USA; 3 California Pacific Medical Center Research Institute, San Francisco, California, USA; 4 Division of the National Toxicology Program, National Institute of Environmental Health Sciences, National Institutes of Health, Department of Health and Human Services, Research Triangle Park, North Carolina, USA; 5 Department of Nutritional Sciences and Toxicology, and; 6 Department of Environmental Health, University of California, Berkeley, Berkeley, California, USA; 7 California Environmental Protection Agency, Sacramento, California, USA; 8 Office of Environmental Health Hazard Assessment, California Environmental Protection Agency, Sacramento, California, USA; 9 Division of Occupational and Environmental Medicine, University of California, San Francisco, San Francisco, California, USA; 10 Natural Resources Defense Council (NRDC), San Francisco, California, USA

## Abstract

**Background:**

Current approaches to chemical screening, prioritization, and assessment are being reenvisioned, driven by innovations in chemical safety testing, new chemical regulations, and demand for information on human and environmental impacts of chemicals. To conceptualize these changes through the lens of a prevalent disease, the Breast Cancer and Chemicals Policy project convened an interdisciplinary expert panel to investigate methods for identifying chemicals that may increase breast cancer risk.

**Methods:**

Based on a review of current evidence, the panel identified key biological processes whose perturbation may alter breast cancer risk. We identified corresponding assays to develop the Hazard Identification Approach for Breast Carcinogens (HIA-BC), a method for detecting chemicals that may raise breast cancer risk. Finally, we conducted a literature-based pilot test of the HIA-BC.

**Results:**

The HIA-BC identifies assays capable of detecting alterations to biological processes relevant to breast cancer, including cellular and molecular events, tissue changes, and factors that alter susceptibility. In the pilot test of the HIA-BC, chemicals associated with breast cancer all demonstrated genotoxic or endocrine activity, but not necessarily both. Significant data gaps persist.

**Conclusions:**

This approach could inform the development of toxicity testing that targets mechanisms relevant to breast cancer, providing a basis for identifying safer chemicals. The study identified important end points not currently evaluated by federal testing programs, including altered mammary gland development, Her2 activation, progesterone receptor activity, prolactin effects, and aspects of estrogen receptor β activity. This approach could be extended to identify the biological processes and screening methods relevant for other common diseases.

**Citation:**

Schwarzman MR, Ackerman JM, Dairkee SH, Fenton SE, Johnson D, Navarro KM, Osborne G, Rudel RA, Solomon GM, Zeise L, Janssen S. 2015. Screening for chemical contributions to breast cancer risk: a case study for chemical safety evaluation. Environ Health Perspect 123:1255–1264; http://dx.doi.org/10.1289/ehp.1408337

## Introduction

Over the last century, synthetic chemicals have become a key material basis of industrialized societies. In 2006, > 34 million metric tons of chemicals were produced in, or imported into, the United States every day, and global chemical production is projected to double over the next 25 years (Wilson and Schwarzman 2009). Hundreds of chemicals are routinely detected in people and in ecosystems worldwide, yet the health and environmental effects of the vast majority of these substances are poorly understood.

To address this information deficiency, chemical hazard evaluation is shifting to emphasize new, more efficient *in vivo* and *in vitro* mechanism-based chemical screening. A 2007 study by the National Academy of Sciences (NAS) concluded that “a transformative paradigm shift is needed” in toxicity testing, one that can detect “upstream events,” that is, early changes in biological processes linked to development of disease (National Research Council 2007). Upstream events most often precede any clinical finding and could be used as early indicators of toxicity. This transformation will involve screening chemicals to detect early indicators of toxicity (e.g., disruption of normal cellular pathways and biological programming) rather than focusing exclusively on observations of apical or overt disease end points, such as the development of a tumor, birth defect, or infertility (National Research Council 2007). In response to the 2007 NAS report, several major federal research initiatives were instigated, including the U.S. Environmental Protection Agency (EPA) ToxCast (Judson et al. 2010), the National Toxicology Program (NTP) High Throughput Screening Initiative (NTP 2015), and the interagency Tox21 Initiative (Schmidt 2009; Tice et al. 2013; NTP 2015). These initiatives are developing rapid, cost-effective methods to screen large numbers of chemicals for toxicity. Questions remain, however, whether high-throughput screening can adequately predict which chemicals will cause endocrine disruption or disease in humans. In particular, many of these programs rely on “off the shelf” commercially available batteries of *in vitro* screens, potentially leaving significant gaps in the assessment of end points relevant to particular tissues or diseases. Important gaps in these assessments may be identified by starting with a disease and working backward through the known and suspected mechanisms associated with the disease. Chemicals that alter these biological processes can be identified through *in vitro* or *in vivo* assays and slated for further testing to determine involvement in disease causality.

A 2013 report by the Interagency Breast Cancer and Environmental Research Coordinating Committee (IBCERCC) described the importance of understanding how and when environmental factors affect biological mechanisms that influence the risk of breast cancer. In other words, they recommended an approach that works backward from a disease to identify the early indicators of toxicity (IBCERCC 2013). In principle, this approach could determine whether reducing or eliminating such chemical exposures could help prevent breast cancer.

Current evidence is inadequate to establish the proportion of breast cancer cases attributable to environmental pollutants. Inherited risk factors by themselves explain only an estimated 5–10%, or at most up to 25%, of breast cancer risk; thus, environmental factors are believed to play an important role in the majority of breast cancers (American Cancer Society 2012; Lichtenstein et al. 2000). Established noninherited causes of breast cancer in humans include exposure to estrogenic compounds [e.g., hormone replacement therapy (HRT) (Chlebowski et al. 2009)], other substances with hormonal effects [e.g., alcohol (Hilakivi-Clarke et al. 2004)], agents that cause direct genetic damage [e.g., ionizing radiation (Brody and Rudel 2008)], and some that act via all of the above mechanisms [e.g., diethylstilbestrol (DES) (Colton et al. 1993; Hoover et al. 2011; IARC 2012d)]. Animal models, however, raise concern for many more chemicals than the few that have been definitively identified as breast carcinogens. More than 200 compounds have been found to induce mammary tumors in animals (Rudel et al. 2007). Of the > 600 chemicals that have been evaluated in adult animals by the NTP in 2-year rodent cancer bioassays, about 60 were determined to cause mammary gland tumors (Macon and Fenton 2013). The evidence is complicated by the fact that carcinogens often have different target organs in different species, such that an agent may cause breast cancer in humans but other, nonmammary cancers in rats (Gold et al. 1991; Haseman and Huff 1987). Furthermore, breast cancer is not a uniform disease, and different classes of chemical carcinogens may raise the risk of different clinical subsets of breast cancer. Close similarities between the molecular profiles of aggressive breast cancers from patients and from nonmalignant human breast cell samples exposed to chemicals under defined *in vitro* conditions (Dairkee et al. 2008) suggest that a wider range of dose and exposure regimens might shed light on the role of environmental chemicals in the genesis of low-risk, indolent breast tumors versus their highly aggressive counterparts.

Chemicals, either alone or in combination with other factors, can act at numerous points in a biological chain of events leading to tumor formation. Although some changes can occur rapidly, in humans the lag time between exposure and disease can be decades. For example, the use of DES is linked to breast cancer and reproductive tract cancers that develop 20–60 years after *in utero* exposure (Hoover et al. 2011; Laronda et al. 2012; Reed and Fenton 2013; Troisi et al. 2007), whereas relatively short latency periods have been observed with HRT (IARC 2012d). Current assessments of potential chemical carcinogens rely on limited human epidemiologic studies, or on laboratory animal studies for evidence of tumor formation. However, laboratory animal studies are expensive, and because they typically expose animals only as adults, and for just a portion of their lives, they do not reflect the impact of developmental exposures or the more typical time lag between exposure and disease in human breast cancer. They are also relatively insensitive to chemicals that contribute to cancer risk indirectly by increasing disease susceptibility.

Increasingly, the agencies, such as the International Agency for Research on Cancer (IARC) and NTP, that assess chemicals for carcinogenicity rely on both animal bioassays and molecular mechanistic evidence to determine whether a chemical is a likely human carcinogen, even in the absence of human data (IARC 2014c; NTP 2013). This approach is consistent with IARC’s definition of a carcinogen as a substance “capable of increasing the incidence of malignant neoplasms, reducing their latency, or increasing their severity or multiplicity” (IARC 2009). Notably, this definition is broad enough to include agents that act indirectly or that promote the growth of tumors initiated by other substances.

Given the tens of thousands of untested chemicals, it is clearly not feasible to run full 2-year cancer bioassays on all chemicals lacking sufficient toxicological information. Thus, the need to distinguish safer from more hazardous chemicals requires rapid, economical screening methods. The U.S. EPA ToxCast (Judson et al. 2010) and the interagency Tox21 (Tice et al. 2013) programs seek to address this by identifying suites of high-throughput tests to accurately characterize chemical hazards. To improve the relevance of this new mechanistic toxicology testing paradigm to human disease, we undertook a study using breast cancer as the outcome and identifying key biological processes associated with the disease. Comparing these processes with those evaluated by both established and emerging toxicological testing approaches reveals important gaps that could be closed with the development of new tests. In addition, the study suggests an approach to chemical testing in which evidence that a chemical alters a biological process linked to breast cancer could trigger further investigation of chemical carcinogenicity even in the absence of evidence from a traditional cancer bioassay.

## Methods

**Figure 1:**
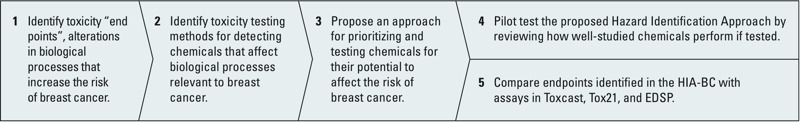
Steps of the breast cancer and chemicals policy project. Abbreviations: EDSP, U.S. EPA Endocrine Disruptor Screening Program; HIA‑BC, Hazard Identification Approach for Breast Carcinogens.

The Breast Cancer and Chemicals Policy (BCCP) project convened an 18 member multidisciplinary expert panel representing the fields of toxicology, cell and molecular biology, cancer models, clinical practice, epidemiology, endocrine disruption, environmental justice, risk assessment, science policy, and breast cancer advocacy (see Supplemental Material, “Breast Cancer and Chemicals Policy Project: Expert Panel”). The panel’s charge was to develop a conceptual strategy for screening chemicals for their potential to cause or contribute to breast cancer in humans. To do this, the panel met over the course of a year to contribute their individual expertise, supplemented by targeted literature searches. Discussions were held in person and remotely, and conclusions were made largely by consensus. The panel approached the charge via the multistep process described below and outlined in Figure 1.

*Step 1. Biological processes associated with breast cancer*. Panel members compiled a catalog of biological alterations strongly associated with breast cancer in the scientific literature, as well as emerging empirical evidence, to create a robust overview of current scientific knowledge of the disease. We designated these alterations “biological processes” based on the definition of a biological process as “operations or sets of molecular events with a defined beginning and end, pertinent to the functioning of integrated living units: cells, tissues, organs, and organisms” (Gene Ontology Consortium 2014). The panel categorized the biological processes under the broad headings of cellular and molecular events, tissue changes, and susceptibility factors associated with the development of, progression of, or susceptibility to breast cancer (Table 1). The panel also identified cellular characteristics associated with cancer but not unique to breast cancer. These “hallmarks of cancer” include changes such as unlimited replication, evasion of apoptosis, and tissue invasion (Hanahan and Weinberg 2000, 2011; Sonnenschein and Soto 2011). We organized the biological processes in spreadsheet form, which became the basis for Step 2.

**Table 1:** Biological processes relevant to breast cancer etiology.

Cellular and molecular events	Tissue changes	Susceptibility factors
Alterations in hormone levels, metabolism, or receptors	Altered mammary gland development	Early onset of puberty
Cell cycle changes	Terminal end bud proliferation	Increased lifetime duration of estrogen exposure (early menarche or late menopause)
Changes in transcription, translation, and epigenetic programming of genes associated with breast cancer	Ductal hyperplasia	Alterations in cyclicity
Altered activity or expression of peptide hormones (growth hormones)	Atypical hyperplasia	Atypical function of metabolizing enzymes
Immune modulation	Increased breast density/stromal hyperplasia	Obesity
Inflammation	Adenomas	
Oxidative stress	Carcinoma *in situ*
Genotoxicity	Tissue invasion^*a*^
Limitless replication potential^*a*^	Sustained/enhanced angiogenesis^*a*^
Evasion of apoptosis^*a*^
Autocrine growth^*a*^
^***a***^Indicators consistent with the hallmarks of cancer progression as defined by Hanahan and Weinberg (2011).		

*Step 2. Toxicity assays*. For each breast cancer–associated biological perturbation identified in Step 1, the panel cataloged existing toxicological assays capable of detecting such changes. Expert judgment, supplemented by targeted literature searches, was used to generate the assay list. We included *in vitro* and *in vivo* assays, as well as human epidemiological studies useful for evaluating the identified perturbations. In addition to validated assays currently available and used by the U.S. EPA Endocrine Disruptor Screening Program (EDSP) (U.S. EPA 2011), U.S. Food and Drug Administration (FDA), NTP, Organisation for Economic Co-operation and Development (OECD), or other established governmental programs, we also included those that could be readily validated in the future. These additional assays included emerging high-throughput toxicity tests and assays used by academic laboratories. The biological processes and test methods were organized into a matrix that served as a working document used in subsequent steps [this working document (BCCP 2010) is available online: http://coeh.berkeley.edu/greenchemistry/cbcrpdocs/matrix.pdf]. Examples of biological processes and assays for detecting perturbations of those processes include

Cell cycle changes assessed by *in vitro* laboratory assays for apoptosis or cell proliferation (Culbreth et al. 2012; Huang et al. 2008)
Hormonal interference causing alterations in female cyclicity determined by estrous status, evaluated by vaginal smears in laboratory animals (Goldman et al. 2007)
Altered mammary gland development; for example, morphological changes evaluated using whole mounts or altered hormone receptor levels in mammary epithelia of animals exposed to chemicals early in life (Fenton 2006; Rudel et al. 2011).

**Figure 2:**
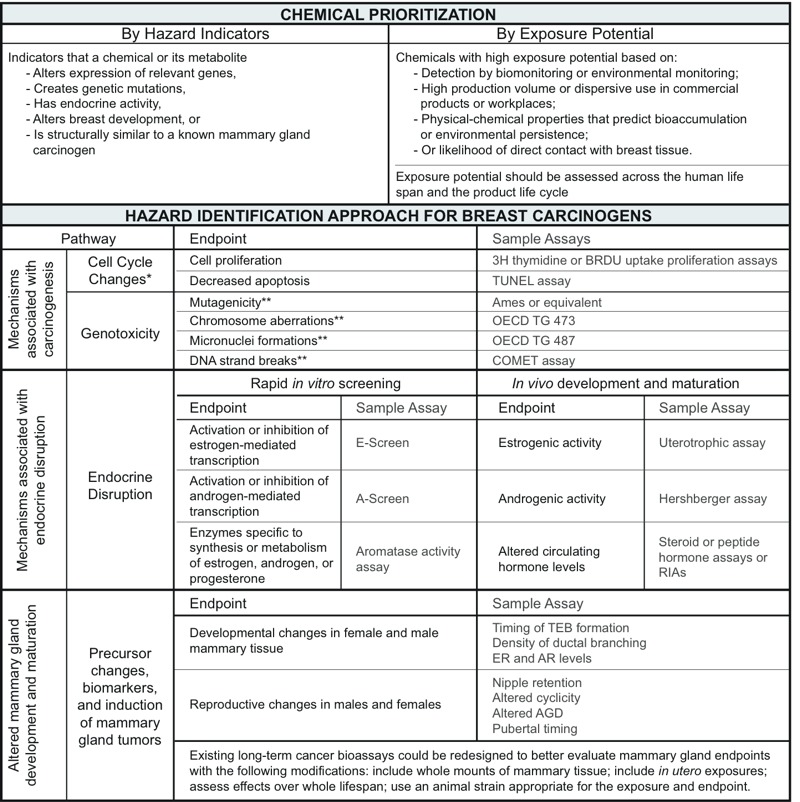
The Hazard Identification Approach to Breast Carcinogens (HIA‑BC). Abbreviations: AGD, anogenital distance; AR, androgen receptor; ER, estrogen receptor; OECD TG 473, OECD Test No. 473: *In Vitro* Mammalian Chromosomal Aberration Test (OECD 2014a); OECD TG 487, OECD Test No. 487: *In Vitro* Mammalian Cell Micronucleus Test (OECD 2014b); RIA, radioimmunoassay; TEB, terminal end bud. *Cell cycle changes are indicators consistent with the hallmarks of cancer progression identified by Hanahan and Weinberg (2000, 2011). **Assessed in mammary epithelial tissue after either *in vitro* or whole animal exposure.

*Step 3. Design of the Hazard Identification Approach for Breast Carcinogens (HIA-BC)*. The panel combined the end points associated with biological processes identified in Step 1 and the associated assays identified in Step 2 to create a testing scheme, the HIA-BC. Rather than highlighting a series of assays that could lose relevance as new test methods emerge, the HIA-BC lists alterations in critical biological processes that should be assessed, providing examples of some assays currently available for detecting such perturbations. The intent is for the HIA-BC to accommodate new assays as test methods evolve. Because of the large number of chemicals that have not been tested, the panel also set criteria for prioritizing chemicals to undergo toxicity testing (Figure 2).

*Step 4. Pilot testing the HIA-BC*. To assess the utility and reliability of the HIA-BC, we conducted a “virtual” pilot test using 11 relatively well-studied chemicals. Chemicals were selected based on IARC cancer classifications, including those known to cause breast cancer in humans (IARC 2014b), those with less than sufficient human evidence of breast cancer, known carcinogens without evidence of causing breast cancer, and well-studied chemicals that have no evidence of carcinogenicity (IARC 2014a). To complete the pilot test, additional chemicals with animal evidence of mammary cancer but no similar human evidence were selected from those identified by Rudel et al. (2007).

**Figure 3:**
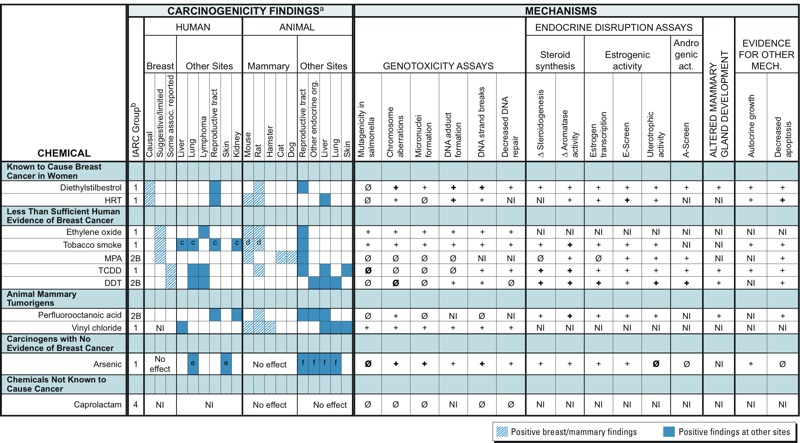
Pilot test of the Hazard Identification Approach for Breast Carcinogens. Results for each chemical in this table are based on references that are included in Supplemental Material, “References for Figure 3, Pilot Test of the HIA‑BC.” Abbreviations: +, Positive finding in a test (irrespective of direction of response), with information from just one or two studies; **+**, positive finding in a test (irrespective of direction of response), with information from a consensus document or that represents a “weight of evidence” (i.e., multiple studies); Ø, a finding of no effect or equivocal evidence; **Ø**, a finding of no effect, with information from a consensus document or that represents a “weight of evidence” (i.e., multiple studies); DDT, dichlorodiphenyltrichloroethane; HRT, hormone replacement therapy (estrogen and progesterone); IARC, International Agency for Research on Cancer; MPA, medroxyprogesterone acetate; NI, not investigated; TCDD, 2,3,7,8-tetrachlorodibenzodioxin. ***^a^***We noted positive findings as reported by IARC. Negative findings or a lack of studies are indicated in cases in which they are relevant to the assignment of a chemical to a category (e.g., “chemicals not known to cause cancer”). ***^b^***IARC classifications: 1, carcinogenic to humans; 2B, possibly carcinogenic to humans; 4, probably not carcinogenic to humans. ***^c^***IARC also identifies causal relationships with tobacco smoke and the following human cancer sites: oral cavity, nasal cavity, pharynx, esophogus, larynx, pancreas, stomach, and bladder. ***^d^***Mammary carcinogenicity in animals demonstrated in tests of constituent chemicals (e.g., benzene and ethylene oxide). ***^e^***IARC also identifies causal relationship with arsenic and bladder cancer. ***^f^***These findings include cancers that occur after prenatal exposure.

We conducted the pilot test as a qualitative assessment of the currently available assays relevant to end points included in the HIA-BC. A literature search identified findings for each of the major categories of mechanisms in the HIA-BC, including end points associated with genotoxicity, endocrine disruption, altered mammary gland development, and other mechanisms representing the hallmarks of cancer, such as autocrine growth and decreased apoptosis. For each chemical, we summarized assay results published for end points that most closely correspond to the biological mechanisms targeted by the HIA-BC. The chemicals, and their performance in assays representing end points in the HIA-BC, are presented in Figure 3. We used the individual compound search tool to search TOXNET, the National Library of Medicine environmental health and toxicology database (NLM 2015). We started with consensus documents and government reports such as IARC monographs, NTP technical reports, Carcinogenesis Research Information Service reports, and Hazardous Substance Data Bank entries. In the absence of consensus documents and government reports, or where IARC evaluations were > 20 years old, we searched PubMed (http://www.ncbi.nlm.nih.gov/pubmed) for the compound name and iterations of the end point of interest. When a PubMed search returned several studies, we summarized the results. If no information was available in any of these sources, we indicated this as “not investigated.”

*Step 5. Comparing breast cancer–relevant end points with new U.S. chemical screening initiatives*. We compared the end points relevant to breast cancer etiology (Table 1) with those included in chemical screening programs under development by the NTP and U.S. EPA to assess how comprehensively those programs evaluate changes in biological processes relevant to breast cancer. To identify assays in ToxCast and Tox21, we conducted a systematic search of assay lists and descriptions published by the two programs (Judson et al. 2010; Kavlock et al. 2012; Tice et al. 2013; U.S. EPA 2013), searching keywords reflecting the biological processes associated with breast cancer identified in Table 1. These include mechanisms associated with cancer in general (genotoxicity, immune response, inflammatory response, oxidative stress, and cell cycle regulation), endocrine effects [estrogen, androgen, progesterone, thyroid, Her2 (human epidermal growth factor receptor 2), and AhR (aryl hydrocarbon receptor), steroidogenesis, aromatase], and hallmarks of cancer (proliferative signaling, growth suppression, limitless replication, apoptosis, angiogenesis, energy regulation, and metastasis). We also searched for assays conducted in breast cells or with proteins isolated from breast cells. Keywords for cancer hallmark processes included those used by Kleinstreuer et al. (2013); a comprehensive list of keywords is included in Supplemental Materials, “Keywords for searches of assays in Tox21 and ToxCast.” A custom R script took two categories of inputs: *a*) assay lists provided by ToxCast (Judson et al. 2010; Kavlock et al. 2012; U.S. EPA 2013) with descriptive information about assays in ToxCast phase 1, ToxCast phase 2, and Tox21; and *b*) a file containing search keywords. This script produced lists of assays matching search terms, which we reviewed manually. Relevant assays identified by this search are summarized in Supplemental Material, Table S1.

## Results

The HIA-BC (Figure 2) was designed to identify chemicals that perturb biological processes relevant to breast cancer. It begins with a prioritization step, narrowing a multitude of chemicals to those with the highest likelihood of affecting breast tissue. These criteria included preliminary hazard indicators (e.g., structural similarity to known carcinogens) and high exposure potential, based on U.S. EPA–defined measures such as high production volume (U.S. EPA 2007) or chemical persistence and bioaccumulation (U.S. EPA 1999). Chemicals that emerge from the prioritization step would then be tested for three categories of end points associated with an increased risk of breast cancer: *a*) mechanisms associated with carcinogenesis in general, including cell cycle changes and genotoxicity (Gray et al. 2009; Hattis et al. 2009); *b*) mechanisms associated with endocrine disruption (Birnbaum and Fenton 2003); and *c*) altered mammary gland development and maturation (Fenton 2006). Endocrine disruption and mechanisms of carcinogenesis, in general, can be assessed by short-term *in vivo* and/or *in vitro* assays. Alterations to mammary gland development can currently be assessed only by *in vivo* studies. The rationale for including each category of biological processes is discussed below.

*Carcinogenesis*. The HIA-BC includes multiple end points for identifying chemicals that act as mutagens or genotoxicants, or that alter cell cycles (Figure 2). Cell cycle changes include increased cell replication, often accompanied by decreased apoptosis. These end points are widely recognized as markers of increased cellularity, potentially initiating limitless cell replication, a hallmark of cancer. Cell cycle changes can be assessed in both *in vitro* and *in vivo* models.

Genotoxicity is induced by chemicals that are mutagenic (agents that increase the rate of mutations) and/or clastogenic (agents that damage DNA structure). Although some genotoxic chemicals are directly clastogenic (e.g., benzene) or DNA reactive, others can act indirectly via complex signaling pathways involving enzymatic activities and DNA replication (Benfenati et al. 2009). Impaired DNA repair is also associated with the development of breast cancer (Blasiak et al. 2004) and is an end point that could be assessed to determine a chemical’s potential to contribute to the disease. Standard genotoxicity test batteries have been adopted by the International Congress for Harmonization Guidelines, the gold standard for assessing compounds used in clinical trials of human subjects (ICH Steering Committee 2008). The revised methods have been incorporated into the FDA (2012) guidance, which recommends testing new drugs or food ingredients for mutagenicity and clastogenicity using three different assays: a test for bacterial reverse gene mutation, and two assays in mammals or mammalian cells, at least one of which should be performed *in vivo*. *In vitro* mammalian cell systems include the metaphase chromosome aberration assay, the micronucleus assay, and the mouse lymphoma assays. *In vivo* assays include analysis of micronuclei in erythrocytes (in blood or bone marrow) and chromosome aberrations in metaphase cells in bone marrow. These methods could be adapted for use in screening for breast carcinogens.

*Endocrine disruption*. Breast cancer risk is influenced by endogenous hormone levels and by exposure to pharmaceutical hormones, including perimenopausal exposure to HRT and *in utero* exposure to DES (Chlebowski et al. 2013; Hoover et al. 2011). It follows that endocrine disruption from other sources may induce similar effects. Exposure to excess estrogen and other hormones during sensitive stages of development has been associated with breast cell proliferation, aberrant tissue growth, and increased incidence of mammary tumors in rodents (Fenton 2006; Russo and Russo 2004). Furthermore, animal models demonstrate that prenatal exposure to steroid hormones increases the likelihood of developing mammary gland tumors following later exposure to a known carcinogen (Lamartiniere et al. 2011; Rudel et al. 2011). An association has also been demonstrated between endogenous hormones or HRT and increased breast cancer risk in humans (Chlebowski et al. 2013). In addition, exposure to the endocrine-disrupting compounds (EDCs) 2,3,7,8-tetrachlorodibenzo-*p*-dioxin (TCDD) (Flaws et al. 1997; Fenton et al. 2002), diochlorodiphenyltrichloroethane (DDT) (Brown and Lamartiniere 1995; Mrema at al. 2013; Snedeker 2001), atrazine (Rayner et al. 2005), bisphenol A (Acevedo et al. 2013; Matsumoto et al. 2004), and cadmium (Johnson et al. 2003) during critical periods of development have been shown to alter mammary gland development in rodents (Fenton 2006).

Based on this evidence, we identified *in vitro* screening methods for assessing perturbations in steroidogenesis, as well as estrogenic and androgenic activity (Figure 2). Some of these methods have been validated for the EDSP. Additional assays, however, would make the EDSP more relevant to breast cancer. For example, the aromatase activity assay provides data on a chemical’s potential to inhibit the catalytic activity of the aromatase protein, but it does not assess changes to the expression of the aromatase gene, which could also disrupt steroidogenesis and which is regulated by a variety of tissue-specific promoters (Chen et al. 2009; Simpson 2004). Similarly, although progesterone and the progesterone receptor play important roles in mammary gland development and breast cancer progression (Obr and Edwards 2012), no progesterone assays have been adopted into EDSP or other mainstream chemical testing paradigms. However, several relevant progesterone assays exist (Svobodová and Cajthaml 2010); for example, Viswanath et al. (2008) described a two-step screening system for identifying (anti)progestin EDCs. The existing progesterone assays could be adapted to screen chemicals for effects specific to breast tissue.

**Figure 4:**
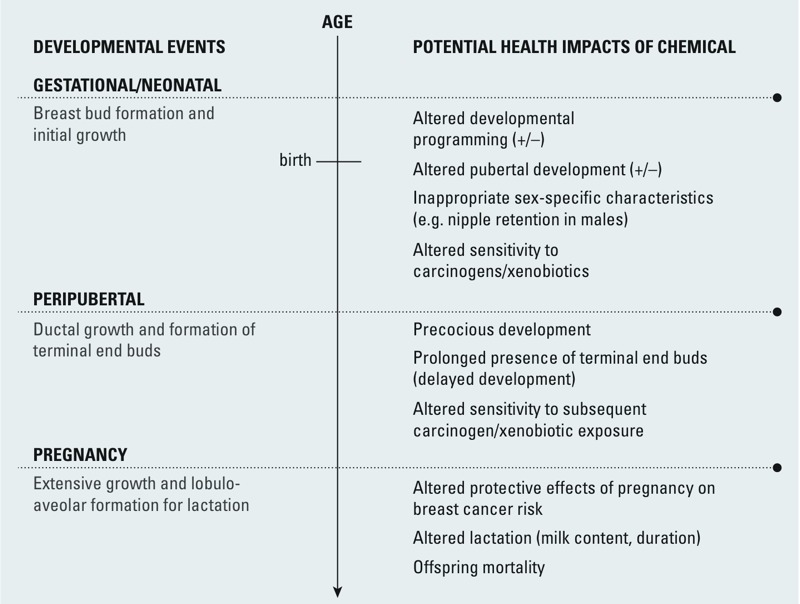
Potential impacts of EDC exposure during critical periods of mammary gland development. Adapted from Fenton (2006). +/–, precocious or delayed.

*Altered development and maturation of the mammary gland*. As discussed above, altering mammary gland development has been shown to alter susceptibility to mammary tumors in rodents (reviewed by Rudel et al. 2011). The mammary gland is highly susceptible to chemical exposures during critical developmental stages, including gestation, puberty, and pregnancy (Figure 4). Until more is known about the molecular processes that govern this pathway, it may be assessed using *in vivo* end points including nipple retention, estrogen receptor (ER) and androgen receptor levels in the gland, and morphological end points such as timing of the development of terminal end buds and other structures, as observed in mammary gland whole mounts (Figure 2).

*Pilot test of the HIA-BC*. The pilot test was intended to assess how well the HIA-BC would perform in identifying breast carcinogens. For substances known to cause breast cancer in women, assays evaluating all of the HIA-BC mechanisms reviewed show positive results. Because both of the substances in this category were designed as synthetic hormones, their endocrine activity is expected. However, genotoxicity is now understood to play a role in DES carcinogenicity and the chemical tests positive in assays of various genotoxicity outcomes (Grosse et al. 2009; IARC 2012d).

A few chemicals have been categorized by IARC as having “less than sufficient” human evidence of increasing breast cancer risk; ethylene oxide is a recognized genotoxic carcinogen (IARC 2012b) but has not been evaluated for endocrine disruption. Tobacco smoke, a complex mixture, shows positive results in many assays, including for genotoxicity (IARC 2012c), endocrine disruption (Kapoor and Jones 2005; Martin et al. 2007), and other cancer hallmarks (IARC 2004). Premenopausal exposure to both direct and secondhand tobacco smoke, particularly before the birth of a first child, is associated with an elevated risk of breast cancer (CA OEHHA 2005; Gaudet et al. 2013). In contrast, medroxyprogesterone acetate (MPA), which has some evidence of increasing breast cancer risk in women (e.g., Li et al. 2012) and is also a mammary carcinogen in multiple animal species, is mostly inactive in genotoxicity assays (IARC 1999a) but is positive in assays for hormone-mediated mechanisms. Among chemicals with weaker evidence of breast cancer in women, DDT (IARC 1991; ATSDR 2002) and TCDD (IARC 2012b) are positive in all of the endocrine assays we evaluated and have negative test results in most of the genotoxicity assays. DDT and TCDD both have reported associations with breast cancer following exposures early in life (around first pregnancy) or long before diagnosis (Cohn et al 2007; Warner et al 2011), and TCDD exposure increases susceptibility to chemically induced mammary tumors in animal studies.

Among chemicals known to be animal mammary tumorigens but that have inadequate human evidence for breast cancer, perfluorooctanoic acid (PFOA) (Benbrahim-Tallaa et al. 2014) tests positive in assays for hormone-mediated mechanisms and is mostly inactive in genotoxicity assays, whereas vinyl chloride, a known genotoxic carcinogen (IARC 2012b), has not been evaluated in the pilot test assays for endocrine effects. Although MPA has only suggestive human evidence of breast carcinogenesis (Li et al. 2012) and the human evidence for PFOA and vinyl chloride is inadequate, the animal mammary evidence could be considered alongside the mechanistic evidence to inform decision making.

In evaluating arsenic, a known human carcinogen not known to cause breast cancer (IARC 2012a), and caprolactam, the only chemical designated by IARC as group 4, “probably not carcinogenic to humans” (IARC 1999b), we attempted to investigate two aspects of the specificity of the HIA-BC: Would a human carcinogen not known to increase breast cancer risk and a chemical not likely to be a carcinogen in any tissue test positive in assays for the end points included in the HIA-BC? Not surprisingly, the known carcinogen arsenic demonstrated genotoxicity. Arsenic was also active in the steroid synthesis and estrogenic activity assays, which could indicate that *a*) other biological activity of arsenic limits its activity in the mammary gland, such as the differential induction of cell death in breast cancer cells (Ruiz-Ramos et al. 2009), or *b*) further epidemiologic study might detect an association between arsenic and breast cancer. Interestingly, arsenic has been investigated as a clinical treatment for advanced breast cancer (Liu et al. 2012; Zhang et al. 2011), and a negative association has recently been observed between exposure to elevated arsenic levels in drinking water and breast cancer mortality in Chile (Smith et al. 2014). Caprolactam emerged from the pilot test without any indications of genotoxicity, but there is no human evidence, and scant data on endocrine disruption or cancer hallmarks.

*Comparing the breast cancer–relevant processes with new U.S. chemical screening initiatives*. There was significant overlap between the end points identified by the panel as relevant for breast cancer and the end points evaluated by assays included in federal chemical screening initiatives (see Supplemental Material, Table S1). ToxCast and Tox21 contain several assays intended to evaluate chemicals’ effects on steroid hormone signaling pathways, including a diverse group of ER end points (ERα and ERβ hetero- and homodimerization, binding, transcriptional activity, and proliferation in an estrogen-dependent breast cancer cell line) (Judson et al. 2010; Rotroff et al. 2013b; Sipes et al. 2013; Tice et al. 2013). They also include many assays reflecting nearly all of the cell behaviors identified as hallmarks of cancer (Hanahan and Weinberg 2011; Judson et al. 2010; Kleinstreuer et al. 2013; Tice et al. 2013), with the exception of limitless replication. Together, ToxCast and Tox21 include assays that measure the activity of aromatase, as well as additional cytochrome p450 enzymes (CYPs) and other enzymes that metabolize estrogens (Judson et al. 2010; Tice et al. 2013). Future plans for ToxCast include the publication of results of assays measuring many intermediates in the steroidogenesis pathway (Judson RS, personal communication).

For some assays, inclusion of breast cells or proteins isolated from them may be important prerequisites to making the assays relevant to breast cancer. ToxCast and Tox21 use systems derived from breast tissue in assays measuring ER, progesterone receptor, androgen receptor, and aromatase activity, as well as cell growth kinetics, cell cycle perturbations, and apoptosis (Rotroff et al. 2013a; Judson et al 2010; Tice et al. 2013). Because of differences in isoforms of CYP and other metabolic enzymes expressed in the breast compared with other organs (e.g., Lehmann and Wagner 2008; Iscan et al. 2001), and because of the tissue-specific nature of many regulatory processes, measurement in breast cells could be also be important for assays measuring gene expression, growth signal regulation, metabolism, and possibly immune and oxidative stress end points.

## Discussion

Based on evidence that a variety of chemicals, especially EDCs, may be contributing to the risk of breast cancer, we designed the HIA-BC to begin addressing the lack of relevant toxicity data by proposing end points useful for screening chemicals for their ability to alter biological processes related to breast cancer. The HIA-BC assembles biological end points associated with increased breast cancer risk, including genotoxicity, endocrine disruption, altered mammary gland development, and some cancer hallmarks. Other general biological processes that may be relevant to breast cancer, such as inflammation, oxidative stress, and immune dysfunction (Table 1), are not currently included in the HIA-BC because of their role in many diseases and lack of specificity to breast cancer, or even to cancer as a whole. However, a comprehensive evaluation of a chemical’s role in breast carcinogenesis might usefully consider disruption of these more general biological processes as well.

For the pilot test of the HIA-BC, we assembled available data for chemicals with a range of evidence, from established human breast carcinogens to a chemical classified as a noncarcinogen. Chemicals associated with breast cancer in either human or rodent studies all demonstrated genotoxic or endocrine disruption, but not necessarily both, although substances with the causal association showed both types of general activity. Among known carcinogens, not all are positive on all genotoxicity assays; for example, DES (IARC 2012d), TCDD (IARC 2012b), and arsenic (IARC 2012a) show negative results for mutagenicity in *Salmonella*. This is to be expected given the variety of mechanisms by which carcinogens can cause tumors to form or grow (Guyton et al. 2009). It is especially significant that some substances, such as MPA, appear to act primarily via endocrine disruption and show no genotoxicity in the end points selected for the pilot. This highlights the many different pathways to carcinogenesis and suggests that a variety of assays is necessary when screening for potential carcinogens.

The final two categories of chemicals included in the pilot test—carcinogens with no evidence of breast cancer and chemicals not known to cause cancer—must be interpreted with caution. Asserting an absence of carcinogenicity, either overall or in breast tissue specifically, presupposes a complete set of test data evaluating all end points relevant to both cancer in general, and breast cancer in particular. Such comprehensive data are unavailable, because of extensive gaps in chemical testing and because of the incompleteness of current scientific understanding of molecular mechanisms associated with breast cancer. Arsenic—an IARC group 1 carcinogen with ample epidemiologic evidence, including prenatal and developmental exposures—served the category of well-studied carcinogens with no convincing evidence of elevated breast cancer risk. In fact, as noted above, protective effects of arsenic on breast cancer mortality have been observed epidemiologically (Smith et al. 2014) and are being explored for therapeutic purposes. Caprolactam—the only chemical designated as IARC group 4, probably not carcinogenic to humans—served the role of a chemical not known to cause cancer. But the absence of testing for endocrine end points makes it difficult to declare this a true negative.

The pilot test also revealed the incompleteness of toxicity testing data for even these relatively well-studied chemicals; almost none had undergone a full battery of published toxicity tests addressing genotoxicity, endocrine disruption, mammary gland development, and cellular behaviors consistent with hallmarks of cancer (e.g., decreased apoptosis). The largest data gaps exist in end points relevant to endocrine disruption and mammary gland developmental effects. As a group, the chemicals lack the full suite of data necessary for understanding their role in breast cancer.

U.S. federal efforts to fill some data gaps are under way in the U.S. EPA’s ToxCast and EDSP and in the interagency Tox21 program. These are research programs designed to develop and validate rapid and predictive mechanistic chemical screening programs. To evaluate the relevance of those testing initiatives for end points associated with breast cancer, we compared the biological processes associated with breast cancer (Table 1) with end points evaluated by assays in ToxCast, EDSP, and Tox21 (see Supplemental Material, Table S1). Although there is significant overlap, the national screening programs could increase their relevance to breast cancer by adding several new end points, including *a*) Her2 activation, *b*) progesterone receptor activity, *c*) prolactin effects, *d*) comprehensive coverage of ERβ activity, and *e*) expression of additional genes that are relevant to breast cancer.

The goal is to move to rapid *in vitro* tests, but some end points such as altered mammary development can still only be assessed *in vivo*. Parallels between rodent and human mammary gland structure and pathology make rodent models useful for characterizing these effects (Fenton 2006). Although further research could better characterize the relationship of rodent mammary gland development to human development, participants at a workshop of > 50 academic and government scientists, half of whose research focuses on mammary gland biology and toxicology, agreed that the rat and mouse are useful models for mammary gland development and carcinogenesis (Rudel et al. 2011). In addition, Rudel et al. (2014) demonstrated high concordance between carcinogenic agents in rodent mammary glands and human breast tissue. Despite these parallels, site concordance across species is not assumed or required when extrapolating from rodents to humans in classifying carcinogens. Ultimately, understanding the molecular mechanisms that drive tissue-level changes seen in altered development should enable the design of improved cell-based assays that help transcend species differences. Because the mammary gland is so vulnerable to altered development from chemical exposures (Rudel et al 2011; Macon and Fenton 2013), it is a priority to develop better *in vitro* tissue models, including systems that model interaction between human epithelial and mesenchymal tissues.

Intended as a screening tool, the HIA-BC should ideally produce more false positives than false negatives. In this respect, the HIA-BC performed well in the pilot test, as no known carcinogens emerged from the screen without testing positive on multiple assays, any of which could serve to flag a chemical for further evaluation. Without a large set of chemicals that have been fully characterized for their potential contribution to breast cancer, true specificity of the HIA-BC is impossible to evaluate. Furthermore, the assays in the pilot test are the closest approximation in the literature for end points stipulated by the HIA-BC. New assays directed at the biological processes specific to breast cancer conducted using test methods relevant to breast tissue might well prove to be more specific to breast cancer than were those whose results are currently available through literature review.

An important goal of this project was to identify key biological processes associated with breast cancer that may not be shared by other target organs. For example, breast cancers appear to be induced by nongenotoxic mechanisms, such as endocrine disruption that alters breast development, as well as genotoxic mechanisms. Yet many chemicals are screened for carcinogenicity using genotoxicity assays alone (e.g., European Chemicals Agency 2014). Screening programs will need to test for these other mechanisms in order to identify all potential breast carcinogens. A similar approach replicated for other tissues and diseases could provide insight into the biological pathways common to many diseases, as well as those that are distinct to a specific disease. Compiled, this information would help ensure that chemical testing initiatives include end points unique to certain diseases, in addition to those that are shared among many disease processes.

Finally, an ideal approach to chemical hazard identification would consist of a set of tiered tests with associated levels of certainty. This would enable stepwise testing of chemicals and facilitate decision making on the basis of limited data. In practice, developing such an approach requires sufficient information on a set of chemicals tested for a full range of end points to be able to assign levels of certainty and create a decision-making algorithm for interpreting test results. The process of attempting to validate the HIA-BC through the pilot test demonstrated that the extent of data gaps in chemical information puts this final step out of reach at the current time. As more data are generated for a larger range of chemicals and end points, this is a critical next step.

## Conclusions

We used an expert panel to identify end points within key biological processes associated with breast cancer and cataloged the assays currently available to evaluate those end points. These are organized into the HIA-BC, an approach to prioritizing and then testing chemicals for their potential to raise the risk of breast cancer. Key end points for screening include DNA damage (genotoxicity), cell cycle changes, endocrine disruption, and altered mammary gland development. Several key biological processes occur only during periods of mammary gland development (Land 1995; Macon and Fenton 2013), so tests need to be carefully designed to capture them. Also, end points that are regulated by tissue-specific mechanisms, such as aromatase transcription (Chen et al. 2009; Simpson 2004), may require tests in mammary tissue models. Altered mammary gland development is an established factor in increased susceptibility to mammary gland tumors, although this end point can currently only be evaluated via *in vivo* tests with prenatal exposure and observation of mammary gland morphology using whole-mounts of the mammary gland (Macon and Fenton 2013; Osborne et al. 2015).

The HIA-BC provides guidance for using existing assays to screen chemicals for their potential role in breast cancer. Although limited by data gaps in the published literature, the pilot test demonstrated that assays for the end points and processes in the HIA-BC would detect carcinogens in general and could also suggest chemicals with the potential to increase breast cancer risk. Further research is needed to *a*) better understand biological processes associated with breast cancer, including those mediated by altered breast development; *b*) develop and validate new assays for processes and end points that are not currently available, including assays suited to high-throughput screening methods; and *c*) investigate cases where using breast tissue–derived cells and proteins would make existing assays more relevant to breast cancer. Further characterization of factors that modulate hormonal activity, genetic polymorphisms that alter hormone metabolism, and the role of epigenetic changes in breast carcinogenesis will contribute to the development of more comprehensive toxicity testing methods. Specific gaps in available test methods for mechanisms associated with breast cancer include progesterone receptor binding (Brisken 2013) and transcriptional activation (Faivre et al. 2008; Kougioumtzi et al. 2014), Her2 activation (Stern 2008), ERβ activity (Pearce and Jordan 2004), and DNA repair mechanisms (Barnes and Camplejohn 1996).

This project provides a model for developing mechanistic chemical screening assays that are relevant to critical disease outcomes. The process used to develop the HIA-BC could be adapted for a range of other diseases, with the ultimate goal of understanding the biological mechanisms common to many disease processes, as well as those that are unique. This understanding could enhance the relevance of new toxicity screening and testing initiatives.

## Supplemental Material

(273 KB) PDF
