# Supplementary material for: Screening for Chemical Contributions to Breast Cancer Risk: A Case Study for Chemical Safety Evaluation
Source: Environ Health Perspect. 2015 Jun 2;123(12):1255–64. doi: 10.1289/ehp.1408337 (PMC4671249; doi:10.1289/ehp.1408337)
Supplement: (273 KB) PDF [file ehp.1408337.s001.acco.pdf]

Environ Health Perspect
DOI: 10.1289/ehp.1408337

Note to Readers: *EHP* strives to ensure that all journal content is accessible to all readers. However, some figures and Supplemental Material published in *EHP* articles may not conform to 508 standards due to the complexity of the information being presented. If you need assistance accessing journal content, please contact ehp508@niehs.nih.gov. Our staff will work with you to assess and meet your accessibility needs within 3 working days.

Supplemental Material

Screening for Chemical Contributions to Breast Cancer Risk: A Case Study for Chemical Safety Evaluation

Megan R. Schwarzman, Janet M. Ackerman, Shanaz H. Dairkee, Suzanne E. Fenton, Dale Johnson, Kathleen M. Navarro, Gwendolyn Osborne, Ruthann A. Rudel, Gina M. Solomon, Lauren Zeise, and Sarah Janssen

Table of Contents

Breast Cancer and Chemicals Policy Project: Expert Panel
Keywords for searches of assays in Tox21 and ToxCast
Table S1. Assays from ToxCast, Tox21, and the EPA Endocrine Disruptor Screening Program that target breast cancer-relevant endpoints
References
References for Figure 3, Pilot Test of the HIA-BC
Diethylstilbestrol
HRT (conjugated equine estrogens + medroxyprogesterone acetate)
Ethylene Oxide
Tobacco Smoke
Medroxyprogesterone Acetate (MPA)
TCDD
DDT
PFOA
Vinyl chloride
Arsenic
Caprolactam

Breast Cancer and Chemicals Policy Project: Expert Panel[†]

<u>**Project Leaders:**</u>

Sarah Janssen, MD, PhD, MPH (Co-Director)
Scientist, Natural Resources Defense Council
Assistant Clinical Professor
Occupational & Environmental Medicine
UC, San Francisco, CA

Megan Schwarzman, MD, MPH (PI and Co-Director)
Center for Occupational and Environmental Health, UC Berkeley; and
Clinical Instructor, Family and Community Medicine
UC, San Francisco, CA

<u>**Panel Members:**</u>

Susan Braun, MA
Executive Director, Commonweal
Bolinas, CA

Vincent James Cogliano, PhD
Head, IARC Monographs Programme
International Agency for Research on Cancer
World Health Organization
Lyon, France

***Shanaz Dairkee, PhD**
California Pacific Medical Center Research Institute
San Francisco, CA

Suzanne Fenton, PhD
Reproductive Endocrinologist
Cellular and Molecular Pathology Branch
National Toxicology Program, NIEHS
Durham, NC

William H. Goodson, III, MD
Senior Clinical Research Scientist
California Pacific Medical Center Research Institute
San Francisco, CA

***Joe Guth, PhD, JD**
Legal Director
Science and Environmental Health Network
Albany, CA

Dale Johnson, PharmD, PhD
Adjunct Professor, Nutritional Science & Toxicology Dept, College of Natural Resources
UC Berkeley, CA

Jean Latimer, PhD
UPCI Hillman Cancer Center
School of Medicine
University of Pittsburgh, PA

Ron Melnick, PhD
Retired Associate, Environmental Toxicology Program
National Institute of Environmental Health Sciences
Research Triangle Park, NC

Rachel Morello-Frosch, PhD, MPH
Associate Professor
Environmental Sciences Policy and Management
College of Natural Resources
UC Berkeley, CA

Ruthann A. Rudel, MS
Senior Scientist, Toxicology and Environmental Health Risk Assessment
Silent Spring Institute
Newton, MA

***Gina Solomon, MD, MPH**
Senior Scientist, Natural Resources Defense Council
Associate Clinical Professor
Occupational and Environmental Medicine and Internal Medicine
UC, San Francisco, CA

Carlos Sonnenschein, MD
Professor, Dept. of Anatomy and Cellular Biology
Tufts University School of Medicine
Boston, MA

***Lauren Zeise, PhD**
Chief, Reproductive and Cancer Hazard Assessment
Office of Environmental Health Hazard Assessment
Cal/EPA Oakland, CA

†Affiliations at the time the project was performed in 2010
*** Member of the core Advisory Panel Group**

Keywords for searches of assays in Tox21 and ToxCast

Searches were case insensitive
Keywords in the same group are separated by

genotoxicity:
mutation|chromosome|chromosomal|micronuclei|micronucleus|adduct|repair|genotox
proliferative signaling: autocrine|growth factor|TGF|insulin|prolifer|EGFR
limitless replication: immortal|telome
invading apoptosis: apoptosis|apopto|BH3
avoiding immune destruction: immune
tumor promoting inflammation: inflamm|sensitization
angiogenesis: angiogenesis|blood vessel|vascul|VEGF|VCAM
energy regulation: energy|hypoxia|mitochondri|glycolysis
metastasis and invasion: metastasis|chemotaxis|cell
adhesion|differentiation|migration|motility|invasion|HGF
growth suppression: suppressor|suppression|CDK|contact

estrogen: estrogen|estradiol|estriol|estrone
steroidogenesis: steroido|HSD|estrone sulfatase|hydroxysteroid dehydrogenase
AHR: ahr|aryl hydrocarbon
aromatase: aromatase|CYP19|CYP-19|CYP 19
androgen: androgen|testosterone|_AR_
thyroid: thyroid
progesterone: progesterone|progestin|progestagen|progestogen|pregnenolone
Her2: Her2|neu |cd340|P185|erbb2

breast: breast|mammary|MCF|MDA-kb2|T47D

cell cycle regulation: cell cycle|mitosis
xenobiotic metabolism:
xenobiotic|monooxygenase|oxidoreductase|transporter|glucuronosyltransferase
oxidative stress: oxidative

Table S1. Assays from ToxCast, Tox21, and the EPA Endocrine Disruptor Screening Program that Target Breast Cancer-Relevant Endpoints.

		ToxCast/Tox21		EDSP
Category	Endpoint/pathway	Assays [a]	In/from breast cells[b]	System
Steroid hormones	Estrogen receptor α	receptor binding/ dimerization/ transcriptional activity; proliferation in estrogen dependent breast cells	yes	in vitro and in vivo
	Estrogen receptor β	*protein dimerization (no receptor binding or transcriptional activity assays)*		[c]
	Androgen receptor	receptor binding/ coactivator recruitment/ transcriptional activity	yes	in vitro and in vivo
	Progesterone receptor	*receptor binding (no transcriptional activity assays)*	yes	
	Aromatase	*inhibition/activity (no expression assays)*	yes	in vitro
	Estrogen metabolism	CYP, SULT, other enzyme activity		
	Steroidogenesis (other)	steroid intermediate levels		in vitro
Other molecular targets involved in endocrine signaling and development	Thyroid receptor	receptor transcriptional activity/ binding		in vivo
	Thyroid (other)	--		
	Her2	--		
	Estrogen-related receptor	*receptor transcriptional activity (no reference compound)*		[c]
	Aryl hydrocarbon receptor	transcriptional activity		
	Prolactin	--		
	Growth factors	growth factor/cytokine (e.g. TGFb, IFN-g) expression; GF receptor (e.g. EGFR) kinase activity and expression [d]		
	PPAR (α, γ, δ)	receptor transcriptional activity/binding		
	ROR	transcriptional activity		
	Epigenetic programming	*histone deacetylation inhibition, mitochondrial function (few or no assays for other epigenetic endpoints)*		
Carcinogenesis	Genotoxicity	*p53, GADD45A levels; HCS DNA damage/ texture (no assays with consistent relationship to standard genotoxicity tests); cytotoxicity in DNA repair deficient cell lines; ATAD5*		
	Inflammation	CEPB, NF-KB activity; OPRL1, P2RY1 binding; CD 40, TNFa, cytokine (e.g. IL1a, IL8,cxcl9, cxcl10, ccl2) levels [d]		
	Xenobiotic metabolizing enzymes	CYP, SULT, UGT, other enzyme activity/expression [d]		
	Cellular stress (including oxidative)	transcriptional activity of stress-related proteins; HCS pH2AX; HCS pcJun [d]		
Other hallmarks of cancer	Evading growth suppressors	p53 levels; HCS pcJun; p38 inhibition [d]		
	Angiogenesis	HIF1a reporter gene assay; proliferation, vascular cell adhesion module (VCAM) in vascular cells; plasmogen activator-related protein (e.g. PLAT, PLAU, PLAUR, SERPINE, THBD) levels [d]		
	Cell cycle changes	mitotic arrest/cell cycle arrest; cell cycle related gene expression	yes	
	Activating tissue invasion and metastasis	cell adhesion/extracellular matrix-related (e.g. ICAM, VCAM, MMP1, PLAT) protein levels; PDE5A activation/inhibition, PPARa/PPARg binding, transcriptional activity [d]		
	Avoiding immune destruction	TCF/b-cat (T-cell specific) transcriptional activity; ISRE transcriptional activity; NF-kB transcriptional activity; proliferation, cytotoxicity, and protein levels in peripheral blood mononuclear cells [d]		
	Evading apoptosis	HCS apoptosis assays; caspase, PDE5, AR inhibition/activity; MYC, p53 protein levels [d]	yes	
	Replicative immortality	--[e]		
Mammary gland development	Morphology	--[f, g]		
	AR, ER in developing MG	--[f]		
Other organism-level endocrine effects	Reproductive development (e.g. AGD, nipple retention, pubertal timing)	--[f]		in vivo
	Circulating hormone levels	--[f]		in vivo

Italics indicate that there is an assay related to the endpoint, but that there are also some gaps.

[a] Several assays in ToxCast provide information about multiple cancer hallmarks; important assays may not be listed under every relevant pathway if they are listed elsewhere in this table.
[b] Indicates if any assays in the category are performed in breast cells, or in the case of cell free assays, using components derived from breast cells.
[c] In vivo estrogenicity tests may reflect some activity from these alternate receptors.
[d] Kleinstreuer et al. (2013) mapped many additional assays to these processes based on target genes' inclusion in Gene Ontology categories matching keywords related to the hallmarks of cancer.
[e] Kleinstreuer et al. (2013) list SIRT2 (involved in chromatin silencing at the telomere) under limitless replication/telomerase.
[f] No in vitro tests exist for these organ and organism-level endpoints, although validated in vivo tests are available.
[g] Protocols for evaluating the morphology of the developing mammary gland are available from the OECD (2008) and have been discussed by Rudel et al. (2011).

References

Kleinstreuer NC, Dix DJ, Houck KA, Kavlock RJ, Knudsen TB, Martin MT, et al. 2013. In vitro perturbations of targets in cancer hallmark processes predict rodent chemical carcinogenesis. Toxicol Sci 131(1):41–55.

OECD (Organisation for Economic Co-operation and Development). 2008. Updated TG 407 Histopathology Guidance Document, Endocrine Disruption: Guidelines for Histological Evaluation, Part 4: Mammay Gland. Available: http://www.oecd.org/chemicalsafety/testing/40613059.pdf [accessed 30 April 2015].

Rudel RA, Fenton SE, Ackerman JM, Euling SY, Makris SL. 2011. Environmental exposures and mammary gland development: state of the science, public health implications, and research recommendations. Environ Health Perspect 119(8):1053–1061.

U.S. EPA. 2013. Archived ToxCast™ Data and Publication Data. Available: http://www.epa.gov/ncct/toxcast/data_archive.html [accessed 30 April 2015].

References for Figure 3, Pilot Test of the HIA-BC

References are organized by chemical, in order of their appearance in Figure 3.

Diethylstilbestrol

Benachour N, Moslemi S, Sipahutar H, Seralini GE. 2007. Cytotoxic effects and aromatase inhibition by xenobiotic endocrine disrupters alone and in combination. Toxicol Appl Pharmacol 222:129-40.

Chen H, Geng CZ, Kuang G, Wang GL, Fan ZL, Wu XD. 2007. Diethylstilbestrol intervention in carcinogenesis of breast cancer in wistar rats. Ai Zheng 26:596-600.

Colerangle JB, Roy D. 1996. Exposure of environmental estrogenic compound nonylphenol to noble rats alters cell kinetics in the mammary gland. Endocrine 4:115-122.

Colerangle JB, Roy D. 1997. Profound effects of the weak environmental estrogen-like chemical bisphenol A on the growth of the mammary gland of Noble rats. J Steroid Biochem Mol Biol 60:153-160.

Doherty L, Bromer J, Zhou Y, Aldad T, Taylor H. 2010. In utero exposure to diesthylstilbesterol (DES) or bisphenol-A (BPA) increases EZH2 expression in the mammary gland: An epigenetic mechanism linking endocrine disruptors to breast cancer. Horm Canc 1:146-155.

DuMond JW, Roy D. 2001. The inhibition of DNA repair capacity by stilbene estrogen in Leydig cells: its implications in the induction of instability in the testicular genome. Mutat Res 483:27-33.

Fielden MR, Fong CJ, Haslam SZ, Zacharewski TR. 2002. Normal mammary gland morphology in pubertal female mice following in utero and lactational exposure to genistein at levels comparable to human dietary exposure. Toxicol Lett 133:181-191.

Geier R, Adler S, Rashid G, and Klein A. 2010. The synthetic estrogen diethylstilbestrol (DES) inhibits the telomerase activity and gene expression of prostate cancer cells. The Prostate 70:1307-1312.

Gell JS, Oh J, Rainey WE, Carr BR. 1998. Effect of estradiol on DHEAS production in the human adrenocortical cell line, H295R. J Soc Gynecol Investig 5:144-8.

Glatt HR, Metzler M, Oesch F. 1979. Diethylstilbestrol and 11 derivatives: a mutagenicity study with Salmonella typhimurium. Mutat Res 67:113-21.

Grosse Y, Baan R, Straif K, Secretan B, El Ghissassi F, Bouvard V, et al. 2009. A review of human carcinogens—Part A: pharmaceuticals. WHO International Agency for Research on Cancer Monograph Working Group. Lancet Oncol 10:13-4.

Gupta C. 2000. The role of estrogen receptor, androgen receptor and growth factors in diethylstilbestrol-induced programming of prostate differentiation. Urol Res 28:223-9.

Hovey RC, Asai-Sato M, Warri A, Terry-Koroma B, Colyn N, Ginsburg E, et al. 2005. Effects of neonatal exposure to diethylstilbestrol, tamoxifen, and toremifene on the BALB/c mouse mammary gland. Biol Reprod 72:423-435.

IARC. 1987. International Agency for Research on Cancer. Overall Evaluations of Carcinogenicity: An Updating of *IARC Monographs* Volumes 1 to 42. IARC Monographs on the Evaluation of Carcinogenic Risks to Humans. Supplement 7. World Health Organization. Lyon, France.

IARC. 2012. International Agency for Research on Cancer. A Review of Human Carcinogens: Pharmaceuticals. IARC Monographs on the Evaluation of Carcinogenic Risks to Humans. Volume 100A. World Health Organization. Lyon, France.

National Toxicology Program. 2002. Current Status of Test Methods for Detecting Endocrine Disruptors: *In Vitro* Estrogen Receptor Transcriptional Activation Assays, NIH Publication No: 03-4505: Background Review Document. Page E-7.

Ninomiya K, Kawaguchi H, Souda M, Taguchi S, Funato M, Umekita Y, et al. 2007. Effects of neonatally administered diethylstilbestrol on induction of mammary carcinomas induced by 7, 12-dimethylbenz(a)anthracene in female rats. Toxicol Pathol 35:813-818.

Odum J, Pyrah IT, Foster JR, Van Miller JP, Joiner RL, Ashby J. 1999. Comparative activities of p-nonylphenol and diethylstilbestrol in noble rat mammary gland and uterotrophic assays. Regul Toxicol Pharmacol 29:184-195.

Odum J, Pyrah IT, Soames AR, Foster JR, Van Miller JP, Joiner RL, et al. 1999. Effects of p-nonylphenol (NP) and diethylstilbestrol (DES) on the Alderley Park (Alpk) rat: comparison of mammary gland and uterus sensitivity following oral gavage or implanted mini-pumps. J Appl Toxicol 19:367-378.

Tomooka Y, Bern HA. 1982. Growth of mouse mammary glands after neonatal sex hormone treatment. J Natl Cancer Inst 69:1347-1352.

Warita, K, Mitsuhashi, T, Sugawara, T, Tabuchi, Y, Tanida, T, Wang, Z-Y, et al. 2010. Direct effects of diethylstilbestrol on the gene expression of the cholesterol side-chain cleavage enzyme (P450scc) in testicular Leydig cells. Life Sci 87:281-5.

Wright SC, Zhong J, Larrick JW. 1994. Inhibition of Apoptosis as a mechanism of tumor promotion. FASEB J 8:654-60.

Yeh S, Miyamoto H, Shima H, Chang C. 1998. From estrogen to androgen receptor: A new pathway for sex hormones in prostate. Proc Natl Acad Sci USA 95:5527-32.

HRT (conjucated equine estrogens + medroxyprogesterone acetate)

Conner P, Register TC, Skoog L, Tani E, von Schoultz B, Cline JM. 2005. Expression of p53 and markers for apoptosis in breast tissue during long-term hormone therapy in cynomolgus monkeys. Am J Obstet Gynecol 193:58-63.

Dinh H, Nathan L. 2002. Medroxyprogesterone acetate does not antagonize estrogen-induced increases in endothelium-dependent vasodilation: potential clinical implications. Fertil Steril 78:122-7.

Franke HR, Vermes I. 2003. Differential effects of progestogens on breast cancer cell lines. Maturitas. 46 Suppl 1:S55-8.

IARC. 2007 International Agency for Research on Cancer. Combined Estrogen-Progestogen Contraceptives and Combined Estrogen-Progestogen Menopausal Therapy. IARC Monographs on the Evaluation of Carcinogenic Risks to Humans. Volume 91. World Health Organization. Lyon, France.

IARC. 2012. International Agency for Research on Cancer. A Review of Human Carcinogens: Pharmaceuticals. IARC Monographs on the Evaluation of Carcinogenic Risks to Humans. Volume 100A. World Health Organization. Lyon, France.

Luo S, Stojanovic M, Labrie C, Labrie F. 1997. Inhibitory Effect Of The Novel Anti-Estrogen Em-800 And Medroxyprogesterone Acetate On Estrone-Stimulated Growth Of Dimethylbenz[A]Anthracene-Induced Mammary Carcinoma In Rats. Int J Cancer 73:580-6.

Mueck AO, Seeger H, Wallwiener D. 2003. Comparison of the proliferative effects of estradiol and conjugated equine estrogens on human breast cancer cells and impact of continuous combined progestogen addition. Climacteric 6:221-7.

Seeger H, Wallwiener D, Mueck AO. 2003. The effect of progesterone and synthetic progestins on serum- and estradiol-stimulated proliferation of human breast cancer cells. Horm Metab Res 35:76-80.

Tseng L, Mazella J, Sun B. 1986. Modulation of Aromatase Activity in Human Endometrial Stromal Cells by Steroids, Tamoxifen and RU 486. Endocrinology 118:1312-8.

Vidal JD, Register TC, Gupta M, Cline JM. 2002. Estrogen replacement therapy induces telomerase RNA expression in the macaque endometrium. Fertil Steril 77:601-8.

Ethylene Oxide

Grosse Y, Baan R, Straif K, Secretan B, El Ghissassi F, Bouvard V, Altieri A, Cogliano V; WHO International Agency for Research on Cancer Monograph Working Group. 2007. Carcinogenicity of 1,3-butadiene, ethylene oxide, vinyl chloride, vinyl fluoride, and vinyl bromide. IARC Monographs on the Evaluation of Carcinogenic Risks to Humans. Volume 97. Lancet Oncol 8:679-80.

IARC. 1994. International Agency for Research on Cancer. Some Industrial Chemicals. IARC Monographs on the Evaluation of Carcinogenic Risks to Humans. Volume 60. World Health Organization. Lyon, France.

IARC. 2012. International Agency for Research on Cancer. A Review of Human Carcinogens: Chemical Agents and Related Occupations. IARC Monographs on the Evaluation of Carcinogenic Risks to Humans. Volume 100F. World Health Organization. Lyon, France.

Pero RW, Widegren B, Högstedt B, Mitelman F. 1981. In vivo and in vitro ethylene oxide exposure of human lymphocytes assessed by chemical stimulation of unscheduled DNA synthesis. Mutat Res 83:271-89.

Steenland K, Whelan E, Deddens J, Stayner L, Ward E. 2003. Ethylene oxide and breast cancer incidence in a cohort study of 7576 women (United States). Cancer Causes Control 14:531-9.

Tobacco Smoke

Barbieri RL, Gochberg J, Ryan KJ. 1986. Nicotine, cotinine, and anabasine inhibit aromatase in human trophoblast in vitro. J Clin Invest 77:1727-33.

Hecht S. 2011. Tobacco Smoke Carcinogens and Lung Cancer. In: Chemical Carcinogenesis (Penning TM, ed). Humana Press, pp 53-74. Available: http://link.springer.com/chapter/10.1007%2F978-1-61737-995-6_3?LI=true#page-1. [accessed 29 April 2015]

IARC. 2012. International Agency for Research on Cancer. A Review of Human Carcinogens: Personal Habits and Indoor Combustions. IARC Monographs on the Evaluation of Carcinogenic Risks to Humans. Volume 100E. World Health Organization. Lyon, France.

IARC. 2004. International Agency for Research on Cancer. Tobacco Smoke and Involuntary Smoking. IARC Monographs on the Evaluation of Carcinogenic Risks to Humans. Volume 83. World Health Organization. Lyon, France.

Kadohama N, Shintani K, Osawa Y. 1993. Tobacco alkaloid derivatives as inhibitors of breast cancer aromatase. Cancer Lett 75:175-82.

Kapoor D, Jones TH. 2005. Smoking and hormones in health and endocrine disorders. Eur J Endocrinol 152:491-9.

Martin MB, Reiter R, Johnson M, Shah MS, Iann MC, Singh B, et al. 2007. Effects of Tobacco Smoke Condensate on Estrogen Receptor-alpha Gene Expression and Activity. Endocrinology 148:4676-86.

Yim HW, Slebos RJ, Randell SH, Umbach DM, Parsons AM, Rivera MP, et al. 2007. Smoking is associated with increased telomerase activity in short-term cultures of human bronchial epithelial cells. Cancer Lett 246:24-33.

Medroxyprogesterone Acetate (MPA)

Franke HR, Vermes I. 2003. Differential effects of progestogens on breast cancer cell lines Maturitas 46:S55-8.

Ghatge RP, Jacobsen BM, Schittone SA, Horwitz KB. 2005. The progestational and androgenic properties of medroxyprogesterone acetate: gene regulatory overlap with dihydrotestosterone in breast cancer cells. Breast Cancer Res 7:R1036-50.

IARC. 1999. International Agency for Research on Cancer. Hormonal Contraception and Post-menopausal Hormonal Therapy. IARC Monographs on the Evaluation of Carcinogenic Risks to Humans. Volume 72. World Health Organization. Lyon, France.

Jeng MH, Parker CJ, Jordan VC. 1992. Estrogenic potential of progestins in oral contraceptives to stimulate human breast cancer cell proliferation. Cancer Res 52:6539-46.

Jordan VC, Jeng MH, Catherino WH, Parker CJ. 1993. The estrogenic activity of synthetic progestins used in oral contraceptives. Cancer 71:1501-5.

Lee TC, Miller WL, Auchus RJ. 1999. Medroxyprogesterone acetate and dexamethasone are competitive inhibitors of different human steroidogenic enzymes. J Clin Endocrinol Metab 84:2104-10.

Lee TC, Miller WL, Auchus RJ. 1999. Medroxyprogesterone acetate (MPA) inhibits pituitary-independent steroidogenesis by inhibiting 3 β-hydroxysteroid dehydrogenase type II (3 β-HSDII). Pediatr Res 45:93A–93A.

Li C, Beaber E, Tang MC, Porter P, Daling JR, Malone K. 2012. Effect of depo-medroxyprogesterone acetate on breast cancer risk among women 20-44 years of age. Cancer Res 72:2028-2035.

Martelli A, Mattioli F, Angiola M, Reimann R, Brambilla G. 2003. Species, sex and inter-individual differences in DNA repair induced by nine sex steroids in primary cultures of rat and human hepatocytes. Mutat Res 536:69-78.

Ory K, Lebeau J, Levalois C, Bishay K, Fouchet P, Allemand I, et al. 2001. Apoptosis inhibition mediated by medroxyprogesterone acetate treatment of breast cancer cell lines. Breast Cancer Res Treat 68:187-98.

Poulin R, Baker D, Poirier D, Labrie F. 1991. Multiple actions of synthetic 'progestins' on the growth of ZR-75-1 human breast cancer cells: an in vitro model for the simultaneous assay of androgen, progestin, estrogen, and glucocorticoid agonistic and antagonistic activities of steroids. Breast Cancer Res Treat 17:197-210.

Rosenfield A, Maine D, Rochat R, Shelton J, Hatcher RA. 1983. The Food and Drug Administration and Medroxyprogesterone Acetate. What Are the Issues? JAMA 249:2922-8.

Seeger H, Wallwiener D, Mueck AO. 2003. Comparison of the effect of progesterone, medroxyprogesterone acetate and norethisterone on the proliferation of human breast cancer cells. J Br Menopause Soc 9:36-8.

Seeger H, Wallwiener D, Mueck AO. 2003. The effect of progesterone and synthetic progestins on serum- and estradiol-stimulated proliferation of human breast cancer cells. Horm Metab Res 35:76-80.

Siddique YH, Afzal M. 2008. A Review on the Genotoxic Effects of Some Synthetic Progestins. Int J Pharmacol 4: 410-430.

Siddique YH, Ara G, Beg T, Afzal M. 2006. Genotoxic potential of medroxyprogesterone acetate in cultured human peripheral blood lymphocytes. Life Sci 80:212-8.

Sonneveld E, Jansen HJ, Riteco JA, Brouwer A, van der Burg B. 2005. Development of Androgen- and Estrogen-Responsive Bioassays, Members of a Panel of Human Cell Line-Based Highly Selective Steroid-Responsive Bioassays. Toxicol Sci 83:136-48.

Tseng L, Mazella J, Sun B. 1986. Modulation of Aromatase Activity in Human Endometrial Stromal Cells by Steroids, Tamoxifen and RU 486. Endocrinology 118:1312-8.

TCDD

Adamsson A, Simanainen U, Viluksela M, Paranko J, Toppari J. 2009. The effects of 2,3,7,8-tetrachlorodibenzo-p-dioxin on foetal male rat steroidogenesis. Int J Androl 32:575-85.

Agency for Toxic Substances and Disease Registry (ATSDR). 1998. Toxicological Profile for Chlorinated Dibenzo-p-dioxins (CDDs). Atlanta, GA: U.S. Department of Health and Human Services, Public Health Service. http://www.atsdr.cdc.gov/toxprofiles/tp104.pdf. [accessed 29 April 2015]

Brown NM, Manzolillo PA, Zhang JX, Wang J, Lamartiniere CA. 1998. Prenatal TCDD and predisposition to mammary cancer in the rat. Carcinogenesis 19:1623-1629.

Brunnberg S, Andersson P, Poellinger L, Hanberg A. 2011. The constitutively active Ah receptor (CA-AhR) mouse as a model for dioxin exposure - effects in reproductive organs. Chemosphere 85:1701-6.

Chan CYY, Kim PM and Winn LM. 2004. TCDD Affects DNA Double Strand-Break Repair. Toxicol. Sci 81: 133-138.

Choi EJ, Toscano DG, Ryan JA, Riedel N, Toscano WA Jr. 1991. Dioxin Induces Transforming Growth Factor-alpha in Human Keratinocytes. J Biol Chem 266:9591-9597.

Fenton SE, Hamm JT, Birnbaum LS, Youngblood GL. 2002. Persistent abnormalities in the rat mammary gland following gestational and lactational exposure to 2,3,7,8-tetrachlorodibenzo-p-dioxin (TCDD). Toxicol Sci 67:63-74.

IARC. 2012. International Agency for Research on Cancer. A Review of Human Carcinogens: Chemical Agents and Related Occupations. IARC Monographs on the Evaluation of Carcinogenic Risks to Humans. Volume 100F. World Health Organization. Lyon, France.

Jenkins S, Rowell C, Wang J, Lamartiniere CA. 2007. Prenatal TCDD exposure predisposes for mammary cancer in rats. Reprod Toxicol 23:391-396.

Katic J, Cemeli E, Baumgartner A, Laubenthal J, Bassano I, Stølevik SB, et al. 2010. Evaluation of the genotoxicity of 10 selected dietary/environmental compounds with the in vitro micronucleus cytokinesis-block assay in an interlaboratory comparison. Food Chem Toxicol 48:2612-23.

Lai KP, Wong MH, Wong CK. 2005. Effects of TCDD in modulating the expression of Sertoli cell secretory products and markers for cell-cell interaction. Toxicology 206:111-23.

Murk AJ, Legler J, Denison MS, Giesy JP, van de Guchte C, Brouwer A. 1996. Chemical-activated luciferase gene expression (CALUX): a novel in vitro bioassay for Ah receptor active compounds in sediments and pore water. Fundam Appl Toxicol 33:149-60.

Takeda T, Taura J, Fujii M, Koga T, Ishii Y, Yamada H. 2011. The effect of maternal exposure to dioxin on fetal steroidogenesis in the steroidogenic organs. Fukuoka Igaku Zasshi 102:159-66.

Vanparys C, Depiereux S, Nadzialek S, Robbens J, Blust R, Kestemont P, et al. 2010. Performance of the flow cytometric E-screen assay in screening estrogenicity of pure compounds and environmental samples. Sci Total Environ 408:4451-60.

Vinggaard AM, Hnida C, Larsen JC. 2000. Environmental polycyclic aromatic hydrocarbons affect androgen receptor activation in vitro. Toxicology 145:173-83.

Vorderstrasse BA, Fenton SE, Bohn AA, Cundiff JA, Lawrence BP. 2004. A novel effect of dioxin: exposure during pregnancy severely impairs mammary gland differentiation. Toxicol Sci 78:248-257.

Warner M, Mocarelli P, Samuels S, Needham L, Brambilla P, Eskenazi B. 2011. Dioxin exposure and cancer risk in the Seveso Women's Health Study. Environ Health Perspect 119:1700-1705.

DDT

ATSDR (Agency for Toxic Substances and Disease Registry). 2002. Toxicological profile for DDT, DDE, DDD. Atlanta, GA: U.S. Department of Health and Human Services, Public Health Service.

Brown NM, Lamartiniere CA. 1995. Xenoestrogens alter mammary gland differentiation and cell proliferation in the rat. Environ Health Perspect 103:708-713.

Chen CW, Hurd C, Vorojeikina DP, Arnold SF, Notides AC . 1997. Transcriptional activation of the human estrogen receptor by DDT isomers and metabolites in yeast and MCF-7 cells. Biochem Pharmacol 53:1161-72.

Clement J, Okey A. 1974. Reproduction in female rats born to DDT-treated parents. Bull Environ Contam Toxicol 12(3):373-377.

Crain DA, Guillette LJ, Rooney AA, Pickford DB. 1997. Alterations in steroidogenesis in alligators (Alligator mississippiensis) exposed naturally and experimentally to environmental contaminants. Environ Health Perspect 105:528-533.

De Flora S, Zanacchi P, Camoirano A, Bennicelli C, and Badolati GS. 1984. Genotoxic activity and potency of 135 compounds in the Ames reversion test and in a bacterial DNA-repair test. Mutat Res 133:161-98.

Duby R, Travis H, Terrill C. 1971. Uterotropic activity of DDT in rats and mink and its influence on reproduction in the rat. Toxicol Appl Pharmacol 18:348-355.

Galindo Reyes JG, Leyva NR, Millan OA, Lazcano GA. 2002. Effects of pesticides on DNA and protein of shrimp larvae Litopenaeus stylirostris of the California Gulf. Ecotoxicol Environ Saf 53:191-5.

Guillette LJ, Gross TS, Gross DA, Rooney AA, Percival HF. 1995. Gonadal steroidogenesis in vitro from juvenile alligators obtained from contaminated or control lakes. Environ Health Perspect 103:31-36.

Hassoun E, Bagchi M, Bagchi D, Stohs SJ. 1993. Comparative studies on lipid peroxidation and DNA-single strand breaks induced by lindane, DDT, chlordane and endrin in rats. Comp Biochem Physiol C 104:427-31.

IARC. 1991. International Agency for Research on Cancer. Occupational Exposures in Insecticide Application, and Some Pesticides. IARC Monographs on the Evaluation of Carcinogenic Risks to Humans. Volume 53. World Health Organization. Lyon, France.

Kelce WR, Stone CR, Laws SC, Gray LE, Kemppainen JA, Wilson EM. 1995. Persistent DDT metabolite p,p'-DDE is a potent androgen receptor antagonist. Nature 375:581-5.

Maness S, McDonnell D, Gaido K. 1998. Inhibition of androgen receptor-dependent transcriptional activity by DDT isomers and methoxychlor in HepG2 human hepotema cells. Toxicol Appl Pharmacol 151:135-42.

Mrema EJ, Rubino FM, Brambilla G, Moretto A, Tsatsakis AM, Colosio C. 2013. Persistent organochlorinated pesticides and mechanisms of their toxicity. Toxicology 307:74-88.

Nelson JA. 1974. Effects of dichlorodiphenyltrichloroethane (DDT) analogs and polychlorinated biphenyl (PCB) mixture on 17β-[3H]estradiol binding to rat uterine receptor. Biochem Pharmacol 23:447-51.

Pérez-Maldonado IN, Díaz-Barriga F, de la Fuente H, González-Amaro R, Calderón J, Yáñez L. 2004. DDT induces apoptosis in human mononuclear cells in vitro and is associated with increased apoptosis in exposed children. Environ Res 94:38-46.

Sanderson JT, Boerma J, Lansbergen GW, van der Berg M. 2002. Induction and inhibition of aromatase activity by various classes of pesticides in H295R human adrenocortical carcinima cells. Toxicol Appl Pharmacol 182:44-54.

Snedeker SM. Pesticides and Breast Cancer Risk: A Review of DDT, DDE, and Dieldrin. 2001. Environ Health Persp 109(S1):35-47.

Sohoni P, Sumpter J. 1998. Several environmental oestrogens are also anti-androgens. J Endocrinol 158:327-39.

Soto AM, Sonnenschein C, Chung KL, Fernandez MF, Olea N, Serrano FO. 1995. The E-SCREEN assay as a tool to identify estrogens: an update on estrogenic environmental pollutants. Environ Health Perspect 103:113-22.

Wójtowicz AK, Milewicz T, Gregoraszczuk EŁ. 2007. DDT and its metabolite DDE alter steroid hormone secretion in human term placental explants by regulation of aromatase activity. Toxicol Lett 173:24-30.

PFOA

Alexander J, Auðunsson GA, Benford D, Cockburn A, Cravedi JP, Dogliotti E, et al. 2008. Perfluorooctane sulfonate (PFOS), perfluorooctanoic acid (PFOA) and their salts: Scientific Opinion of the Panel on Contaminants in the Food chain. The EFSA Journal 653:1-131. Available: http://www.efsa.europa.eu/de/scdocs/doc/653.pdf [accessed 29 April 2015].

Bernbrahim-Talla L, Lauby-Secretan B, Loomis D, Guyton K, Grosse Y, Ghissassi FE, Bouvard V, Guha N, Mattock H, Straif K. 2014. Carcinogenicity of perfluorooctanoic acid, tetrafluoroethylene, dichloromethane, 1,2-dichloropropane, and 1,3-propane sultone. Lancet Onc 15:924-925.

Dixon D, Reed CE, Moore AB, Gibbs-Flournoy EA, Hines EP, Wallace EA, et al. 2012. Histopathologic changes in the uterus, cervix and vagina of immature CD-1 mice exposed to low doses of perfluorooctanoic acid (PFOA) in a uterotrophic assay. Reprod Toxicol 33:506-12.

Henry ND, Fair PA. 2013. Comparison of in vitro cytotoxicity, estrogenicity and anti-estrogenicity of triclosan, perfluorooctane sulfonate and perfluorooctanoic acid. J Appl Toxicol 33:265-72.

Klaunig JE, Hocevar BA, Kamendulis LM. 2012. Mode of Action analysis of perfluorooctanoic acid (PFOA) tumorigenicity and Human Relevance. Reprod Toxicol 33:410-8.

Kraugerud M, Zimmer KE, Ropstad E, Verhaegen S. 2011. Perfluorinated compounds differentially affect steroidogenesis and viability in the human adrenocortical carcinoma (H295R) in vitro cell assay. Toxicol Lett 205:62-8.

NTP. Perfluorooctanoic Acid - M910070. Available: http://ntp.niehs.nih.gov/testing/status/agents/ts-m910070.html [accessed 6 May 2015]

Takagi A., Sai K., Umemura T, Hasegawa R, Kurokawa Y. Short-term exposure to the peroxisome proliferators, perfluorooctanoic acid and perfluorodecanoic acid, causes significant increase of 8-hydroxydeoxyguanosine in liver DNA of rats. Cancer Lett. 1991 Apr;57(1):55-60.

White SS, Calafat AM, Kuklenyik Z, Villanueva L, Zehr RD, Helfant L, et al. 2007. Gestational PFOA exposure of mice is associated with altered mammary gland development in dams and female offspring. Toxicol Sci 96:133-144.

White SS, Kato K, Jia LT, Basden BJ, Calafat AM, Hines EP, et al. 2009. Effects of perfluorooctanoic acid on mouse mammary gland development and differentiation resulting from cross-foster and restricted gestational exposures. Reprod Toxicol 27:289-298.

White SS, Stanko JP, Kato K, Calafat AM, Hines EP, Fenton SE. 2011. Gestational and chronic low-dose PFOA exposures and mammary gland growth and differentiation in three generations of CD-1 mice. Environ Health Perspect 119:1070-1076.

Yang C, Tan YS, Harkema JR, Haslam SZ. 2009. Differential effects of peripubertal exposure to perfluorooctanoic acid on mammary gland development in C57Bl/6 and Balb/c mouse strains. Reprod Toxicol 27:299-306.

Yao X, Zhong L. 2005. Genotoxic risk and oxidative DNA damage in HepG2 cells exposed to perfluorooctanoic acid. Mutat Res 587:38-44.

Vinyl chloride

IARC. 2008. International Agency for Research on Cancer. 1,3-Butadiene, Ethylene Oxide and Vinyl Halides (Vinyl Fluoride, Vinyl Chloride and Vinyl Bromide). IARC Monographs on the Evaluation of Carcinogenic Risks to Humans. Volume 97. World Health Organization. Lyon, France.

IARC. 2012. International Agency for Research on Cancer. A Review of Human Carcinogens: Chemical Agents and Related Occupations. IARC Monographs on the Evaluation of Carcinogenic Risks to Humans. Volume 100F. World Health Organization. Lyon, France.

Swenberg JA, Bogdanffy MS, Ham A, Holt S, Kim A, Morinello EJ, et al. 1999. Formation and repair of DNA adducts in vinyl chloride- and vinyl fluoride-induced carcinogenesis. IARC Sci Publ 150:29-43.

U.S. Environmental Protection Agency (US EPA). 2000. Toxicological Review Of Vinyl Chloride (Cas No. 75-01-4): In Support Of Summary Information On The Integrated Risk Information System (IRIS). Washington, D.C. EPA/635R-00/004.

Arsenic

Akram Z, Jalali S, Shami SA, Ahmad L, Batool S, Kalsoom O. 2010. Adverse effects of arsenic exposure on uterine function and structure in female rat. Exp Toxicol Pathol 62:451-9.

Chang SI, Jin B, Youn P, Park C, Park JD, Ryu DY. 2007. Arsenic-induced toxicity and the protective role of ascorbic acid in mouse testis. Toxicol Appl Pharmacol 218:196-203.

Chattopadhyay S, Pal Ghosh S, Ghosh D, Debnath J. 2003. Effect of dietary co-administration of sodium selenite on sodium arsenite-induced ovarian and uterine disorders in mature albino rats. Toxicol Sci 75:412-22.

IARC. 2004. International Agency for Research on Cancer. Some Drinking-water Disinfectants and Contaminants, including Arsenic. IARC Monographs on the Evaluation of Carcinogenic Risks to Humans. Volume 84. World Health Organization. Lyon, France.

IARC. 2012. International Agency for Research on Cancer. A Review of Human Carcinogens: Arsenic, Metals, Fibres, and Dusts. IARC Monographs on the Evaluation of Carcinogenic Risks to Humans. Volume 100C. World Health Organization. Lyon, France.

Mo J, Xia Y, Ning Z, Wade TJ, Mumford JL. 2009. Elevated human telomerase reverse transcriptase gene expression in blood cells associated with chronic arsenic exposure in Inner Mongolia, China. Environ Health Perspect 117:354-60.

OEHHA. 2004. Public Health Goals for Chemicals in Drinking Water: Arsenic. Sacramento, CA: Office of Environmental Health Hazard Assessment, California Environmental Protection Agency.

Rosenblatt AE, Burnstein KL. 2009. Inhibition of androgen receptor transcriptional activity as a novel mechanism of action of arsenic. Mol Endocrinol 23:412-21.

Shen J, Liu J, Xie Y, Diwan BA, Waalkes MP. 2007. Fetal onset of aberrant gene expression relevant to pulmonary carcinogenesis in lung adenocarcinoma development induced by in utero arsenic exposure. Toxicol Sci 95:313-20.

Smith AH, Marshall G, Yuan Y, Steinmaus C, Liaw J, Smith MT, et al. 2014. Rapid Reduction in Breast Cancer Mortality with Inorganic Arsenic in Drinking Water. EBioMedicine 1 (1): 58-63.

Stoica A, Pentecost E, Martin MB. 2000. Effects of Arsenite on Estrogen Receptor-alpha Expression and Activity in MCF-7 Breast Cancer Cells. Endocrinology 141: 3595–3602.

Wang TS, Hsu TY, Chung CH, Wang AS, Bau DT, Jan KY. 2001. Arsenite induces oxidative DNA adducts and DNA-protein cross-links in mammalian cells. Free Radic Biol Med 31:321-30.

Yih LH, Tseng YY, Wu YC, Lee TC. 2006. Induction of centrosome amplification during arsenite-induced mitotic arrest in CGL-2 cells. Cancer Res 66:2098-106.

Caprolactam

IARC. 1999. International Agency for Research on Cancer. Re-evaluation of Some Organic Chemicals, Hydrazine and Hydrogen Peroxide. IARC Monographs on the Evaluation of Carcinogenic Risks to Humans. Volume 71. World Health Organization. Lyon, France.

Williams GM, Mori H, McQueen CA. 1989. Structure-activity relationships in the rat hepatocyte DNA-repair test for 300 chemicals. Mutat Res 221:263-86.
